# Accelerated elastin degradation by age-disease interaction: a common feature in age-related diseases

**DOI:** 10.1038/s41514-024-00143-7

**Published:** 2024-02-27

**Authors:** Naomi Shek, Anna-Maria Choy, Chim C. Lang, Bruce E. Miller, Ruth Tal-Singer, Charlotte E. Bolton, Neil C. Thomson, James D. Chalmers, Matt J. Bown, David E. Newby, Faisel Khan, Jeffrey T. J. Huang

**Affiliations:** 1 Systems Medicine, School of Medicine, University of Dundee, Dundee, UK; 2 Molecular and Clinical Medicine, School of Medicine, University of Dundee, Dundee, UK; 3 COPD Foundation, Miami, USA; 4 Global Allergy and Airways Patient Platform, Vienna, Austria; 5 Centre for Respiratory Research, NIHR Nottingham Biomedical Research Centre, Translational Medical Sciences, School of Medicine, University of Nottingham, Nottingham, UK; 6 School of Infection and immunity, University of Glasgow, Glasgow, Scotland UK; 7 Department of Cardiovascular Sciences and NIHR Leicester Biomedical Research Centre, University of Leicester, Leicester, UK; 8 MRC / University of Edinburgh Centre for Inflammation Research, Queen’s Medical Research Institute, Edinburgh, Scotland UK

**Keywords:** Cardiovascular diseases, Biochemistry

## Abstract

Aging is a major driving force for many diseases but the relationship between chronological age, the aging process and age-related diseases is not fully understood. Fragmentation and loss of ultra-long-lived elastin are key features in aging and several age-related diseases leading to increased mortality. By comparing the relationship between age and elastin turnover with healthy volunteers, we show that accelerated elastin turnover by age-disease interaction is a common feature of age-related diseases.

## Introduction

Advanced age exponentially increases the risk of developing cardiovascular disease, chronic respiratory diseases including COPD and bronchiectasis, and degenerative diseases such as arthritis^1^. Around 100,000 people worldwide die each day due to age-related diseases^2^. Loss of tissue and structural integrity leading to the loss physiological integrity with age, resulting in a progressive decline of homoeostasis and reduced capacity to respond to environmental stimuli, is believed to contribute to an incremental risk and severity of these diseases^3^. Several intertwined biological mechanisms including shortened telomeres, epigenetics, inflammation, macromolecular damage, altered metabolism and proteostasis, and reduced stem cells and regeneration have been proposed as key mechanisms driving the loss of physiological integrity in aging and its associated diseases^4^. Although most studies have focused on the associations between these processes and chronological age or diseases, less attention has been drawn to their interactions where the effect of chronological age on an aging process depends on the presence of a disease.

Ultra-long lived proteins such as elastin, collagen, and eye lens crystalline have been considered as the Achilles heel of the aging proteome^5^ as their damages and losses are not easily repaired. Among them, elastin is unique in providing the characteristics of elasticity, resilience, and deformability of tissues such as the aorta, lung, and skin etc, and its fragmentation and degradation represents an important feature of normal aging^6^. Elastin is a crosslinked polymeric network of tropoelastin monomers catalysed by lysine oxidase during development. In adult tissues, elastin has an extremely low turnover rate with a half-life of ~74 years under normal conditions^7^ in contrast to minutes to days for most intracellular proteins^8^. In general, adult tissues lack the capability of regenerating functional elastic fibre^9^. These two unique properties imply that an increased turnover of this ultra-long-lived protein in adult tissues could result in irreversible changes to elastin-rich tissues^5^. Elastin breakdown products themselves are also known to possess biological activities such as chemotactism, angiogenesis, and inflammatory responses^10^. Monitoring the activity of elastin degradation therefore could be highly relevant to better understanding aging and aging-related diseases.

We have previously investigated the activity of elastin degradation in several age-associated diseases by evaluating the concentrations of circulating total desmosine and isodesmosine (termed cDES), two crosslinking molecules unique to functional elastin^11–14^. We found that cDES concentrations were positively associated with chronological age in patients with COPD^15^, bronchiectasis^12,14^, abdominal aortic aneurysm (AAA)^13^, acute myocardial infarction^16^, and in control participants with normal lung function and free of significant diseases^15^. The elevated cDES concentrations were significantly correlated with higher mortality and cardiovascular morbidity or mortality in these conditions^11–15^. Interestingly, the activity of elastin degradation was accelerated by an age-disease interaction in COPD^11^, supporting the concept that COPD is a disease of accelerated aging. However, whether a similar age-disease interaction also drives elastin degradation in other age-related diseases is unknown. We set out to investigate whether the age-associated elastin degradation is enhanced in AAA, bronchiectasis, rheumatoid arthritis (RA) and COPD compared to control subjects.

**Fig. 1:**
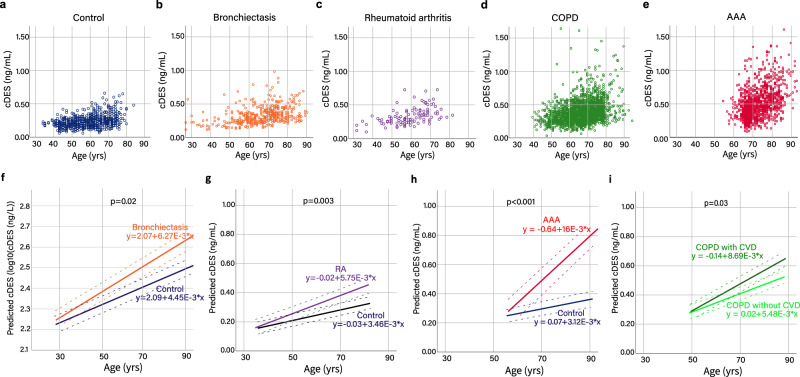
Age-dependent elastin degradation was accelerated in COPD, AAA, bronchiectasis, and rheumatoid arthritis. **a**–**e** Scatterplots of cDES against age in control subjects without inflammatory diseases **a**, bronchiectasis **b**, RA **c**, COPD **d**, AAA **e**. **f** Predicted linear mixed model of log transformed cDES levels and age in bronchiectasis and the control group (β = 0.0048, standard error (SE) = 0.0020, *p* = 0.02, linear mixed effects model for age and disease interaction). Note log10(cDES) was used to ensure normality assumption for residuals and the unit is in ng/L. **g** Predicted linear mixed model of cDES levels and age in RA and the control group (β=0.0009, SE = 0.0003, *p* = 0.003, linear mixed effects model for age and disease interaction). **h** Predicted linear mixed model of cDES levels and age in AAA (β = 0.0030, SE = 0.0009, *p* < 0.001, linear mixed effects model for the age and disease interaction). **i** Predicted linear mixed model of cDES levels in COPD with (*n* = 507) or without CVDs (*n* = 472)( β= 0.0026, SE = 0.0013, *p* = 0.03, linear mixed effects model for age and disease interaction) in the ECLIPSE cohort. Solid regression lines are labelled with equations. Dashed lines represent standard deviations.

We performed a pooled analysis of data from multiple cohorts comprising individuals with COPD (*n* = 1332), AAA (*n* = 507), bronchiectasis (*n* = 433) or RA (*n* = 111), and a control group (*n* = 641). The COPD group was combined from three observational cohorts (the ECLIPSE^17^, Nottingham^17^, and Scotland cohorts^18^) where individuals with GOLD stage II–IV COPD were recruited. The AAA group included two cohorts (the MA^3^RS^19^ and UK Aneurysm Growth Study (UKAGS) cohorts^13^) where the mean AAA size was 48 mm (standard deviation=8 mm). The bronchiectasis patients were from the TAYBRIDGE cohort and had a median (IQR) Bronchiectasis Severity Index score of 6(4–10)^14^. RA patients meeting the 1987 American College of Rheumatology (ACR) classification criteria for RA (DAS28 score = 4.6 (1.6)) were recruited in Ninewells Hospital, Dundee. With the exception of RA and control subjects, previous history of CV disease, uncontrolled hypertension or hypercholesterolaemia and any other inflammatory conditions were not excluded in disease groups. The demographic details of each study group are shown in Supplementary Table 1. The AAA group had a higher percentage of male participants whereas the bronchiectasis and RA groups had higher percentages of females, reflecting the sex differences in the prevalence of those diseases and the fact that only men are screened for AAA in the UKAGS cohort^20,21^. As expected, the COPD and AAA groups have higher percentages of current and ex-smokers. Our previous studies have shown that sex and smoking history did not affect cDES concentrations in adults^12,15^. Similar to the positive associations to age observed in the COPD and control populations^11^, cDES concentrations correlated with age in AAA, bronchiectasis, and RA (*r* = 0.24–0.54, *p* < 0.001, Pearson/Spearman correlation, Fig. 1a–e, Supplementary Table 2).

To investigate the effect of age-disease interaction on elastin turnover, we compared the slopes of the regression lines between the four disease groups and the control population using a linear mixed-effects model incorporating subject-specific random effects and taking into account the effects of multiple visits from some individuals (i.e., in the ECLIPSE, Scotland, and MA^3^RS cohorts). We found that the slope of age to elastin turnover regression in bronchiectasis was greater compared to healthy control subjects (6.27 vs 4.45 pg L^-1^ (log10) per year, *p* = 0.02, linear mixed effects model for the interaction, Fig. 1f). A similar effect of age-disease interaction was found in RA (5.75 vs 3.46 ng L^-1^ per year, *p* = 0.003, linear mixed effects model, Fig. 1g). In the AAA group the predicted regression slopes is ~5 times greater (16 ng L^-1^ per year) when compared to the control population (3.12 ng L^-1^ per year; *p* < 0.001; linear mixed effects model, Fig. 1h). The difference remained if those less than 60 years old (defined by the lower limit of 95% interval of the AAA group) in the control group were excluded (*p* = 0.007, linear mixed effects model). Together with the previous observation in COPD^11^, these results strongly suggest that RA, AAA, bronchiectasis, and COPD are diseases of accelerated elastin turnover driven by an age-disease interaction where the differences between the disease groups and control becomes greater as age increases.

Increased cDES concentrations have implications for mortality in COPD^15^, bronchiectasis^14^, and AAA^13^. Notably, the association between cDES concentrations and cardiovascular diseases was particularly strong among these diseases, probably explained by the abundant presence of elastin in the vascular system. Indeed, a significant interaction (greater slope) was also observed in COPD patients with cardiovascular diseases (defined as self-reported history of hypertension, myocardial infarction, angina, and stroke) compared to COPD participants without in the ECLIPSE cohort (8.69 ng vs 5.48 ng L^-1^ per year, *p* = 0.03, linear mixed effects model, Fig. 1i). The notion is also supported by the fact that among the four diseases studied, AAA showed the greatest slope, possibly reflecting the severe nature of elastin degradation^22^ and the high cardiovascular risk known for this group of patients^23^. Furthermore, cDES concentrations were significantly associated with pulse wave velocity (a measure of arterial stiffness) in patients with COPD^15^ and acute myocardial infarction^16^ further strengthening its clinical relevance to the cardiovascular system with elastin breakdown mirroring arterial stiffness. These data may provide a potential explanation as to why cardiovascular morbidities and mortality are higher in COPD, RA, and bronchiectasis.

It is unclear how this observed age-disease interactions occur across these diseases caused by different aetiologies. It is plausible that old age increases the expansion of blood cells with somatic mutations (or clonal haematopoiesis)^24^, leading to over-activation of immune cells such as neutrophils and macrophages to disease-causing pathogens or reactants^25^, subsequently releasing more proteolytic enzymes to the circulation that digest elastin in the vasculature. Another possibility previously suggested by skin aging studies is that intrinsic aging processes could make elastin more vulnerable over time to elastases stimulated by an extrinsic stimulus or a disease (6). In addition, age associated decline of renal function might also influence the elimination of cDES and cDES containing peptides from the circulation leading to higher cDES concentrations, although our current evidence did not show consistency. In our two previous studies where renal function data are available, we found an association between renal function and cDES concentrations in acute myocardial infarction^16^ but not in COPD^15^. The reason for this discrepancy is unclear but it is possible that the disease population or patient conditions (e.g., acute vs stable condition) could affect the relationship.

Few proven interventions are available that prevent or minimise elastin degradation in humans. To date, alpha-1 antitrypsin augmentation is the only treatment showing the ability of reducing circulating desmosine levels in a randomised trial although the magnitude of treatment effect was small^26^ and it is currently only relevant to alpha-1 antitrypsin deficiency, a rare genetic condition that is associated with emphysema. Next generation systematic anti-proteinase therapy such as DPP1 inhibitors may offer promise especially for bronchiectasis^27^. It would be of great interest to test whether anti-aging therapies such as calorie restriction and caloric restriction mimetics (e.g., metformin) could impact elastin degradation in age-related diseases in human. A recent animal study showing that calorie restriction was able to reduce arterial aging by reducing proinflammation associated elastin degradation supports this concept^28^.

We acknowledge several limitations in this study. First, we were not able to estimate the contribution of renal function to the observed age-disease interaction due to the lack of data availability. Second, the comorbidity data were not always available and therefore the influence of the comorbidity cannot be always excluded. Third, we did not include the acute myocardial infarction study^16^ in the current study as the data were not available.

In summary, we conclude that accelerated elastin degradation by age-disease interaction is a common feature in age-related diseases.

## Methods

### Clinical cohorts

Multiple cohorts comprising individuals with COPD, AAA, bronchiectasis or RA, and a control group were included in this study. The COPD group was combined from three observational cohorts (the ECLIPSE^17^(ClinicalTrials.gov Identifier: NCT00292552), Nottingham^17^, and Scotland cohorts^18^). The AAA group included two cohorts (the MA^3^RS^19^(ClinicalTrials.gov Identifier: NCT02229006) and UKAGS cohorts^13^). The bronchiectasis patients were from the TAYBRIDGE cohort^14^. RA patients meeting the 1987 ACR classification criteria for RA were recruited in Ninewells Hospital, Dundee. Ethic approvals and informed consent have been obtained and the details can be found in previous reports^13,14,17–19^.

### cDES quantification

cDES concentrations were measured using the same validated isotope dilution LC-MS/MS method^29^ in the same laboratory.

### Statistical analysis

Correlation analysis and linear mixed-effects modelling were carried out using SPSS (version 25, IBM). For linear mixed modelling, the fixed effects included age, disease groups (e.g., COPD vs control), as well as the interaction between age and disease groups. Effects from repeated measurements were controlled for. The random effects included intercept and/or age using two covariance structures (variance components or unstructured). The Akaike information criterion was utilised to select the best valid model that yielded the lowest AIC. The normality of residuals was checked by Q-Q plots and the homogeneity of variance by scatter plots of residuals and predicted values. A *p* value of 0.05 was considered significant.

### Reporting summary

Further information on research design is available in the Nature Research Reporting Summary linked to this article.

### Supplementary information

Supplementary Information

Reporting Summary

## Data Availability

The clinical data for the ECLIPSE cohort is available at dbGaP (Study Accession: phs001252.v1.p1). Other data are available from the corresponding author on reasonable request.
