# Supplementary material for: Accelerated elastin degradation by age-disease interaction: a common feature in age-related diseases
Source: NPJ Aging. 2024 Feb 27;10(1):15. doi: 10.1038/s41514-024-00143-7 (PMC10899634; doi:10.1038/s41514-024-00143-7)
Supplement: Supplementary file 1 — Supplementary Information [file 41514_2024_143_MOESM1_ESM.pdf]

Supplementary Information

Accelerated elastin degradation by age-disease interaction: a common feature in age-related diseases

Naomi Shek[1], Anna-Maria Choy[2], Chim C Lang[2], Bruce E Miller[3], Ruth Tal-Singer[4], Charlotte E Bolton[5], Neil C Thomson[6], James D Chalmers[2], Matt J Bown[7], David E Newby[8], Faisel Khan[1], and Jeffrey TJ Huang[1]

1. Systems Medicine, School of Medicine, University of Dundee, Dundee DD1 9SY, United Kingdom
2. Molecular and Clinical Medicine, School of Medicine, University of Dundee, Dundee DD1 9SY, United Kingdom
3. COPD Foundation, USA
4. Global Allergy and Airways Patient Platform, Vienna Austria
5. Centre for Respiratory Research, NIHR Nottingham Biomedical Research Centre, Translational Medical Sciences, School of Medicine, University of Nottingham, Nottingham, United Kingdom
6. School of Infection and immunity, University of Glasgow, Glasgow, Scotland, United Kingdom
7. Department of Cardiovascular Sciences and NIHR Leicester Biomedical Research Centre, University of Leicester, Leicester, United Kingdom
8. MRC / University of Edinburgh Centre for Inflammation Research, Queen's Medical Research Institute, 47 Little France Crescent, Edinburgh EH16 4TJ, Scotland, United Kingdom

Correspondence: Dr Jeffrey T.-J. Huang, Systems Medicine, School of Medicine, University of Dundee, Dundee DD1 9SY, United Kingdom. Tel: +44 (0)1382 386901; e-mail: jtjhuang@dundee.ac.uk

Supplementary Table 1 – Baseline characteristics of study participants

	Control	COPD	AAA	Bronchiectasis	RA
**Number of participants**	641	1332	507	433	111
**Age**	58±10	65±8	72±6	67±8	59±10
**Sex M/F (% men)**	327/314 (51)	815/517 (61)	477/30 (94)	170/263 (39)	34/77 (31)
**Current smokers (%)**	37	27	21	4	20
**Former smokers (%)**	22	73	76	35	N/A
**COPD (%)**	0	100	13.6	5.1	0
**Body mass index kg/m^2**	27±5	27±6	28±4	25±7	26±5
**Hypertension (%)**	22	35	64	n.a.	25
**Stroke (%)**	1	4	7	n.a.	0
**Prior myocardial infarction (%)**	2	10	25	n.a.	0
**Diabetes (%)**	5	10	17	n.a.	0
**RA (%)**	0	0	n.a.	5	100

n.a. = not available.

Supplementary Table 2 – Correlation between cDES levels and age by disease groups

	rho (uncorrected, Pearson/Spearman)[1]	p-value (uncorrected)	rho (predicted)[2]	p-value (predicted)
Control	0.36/0.34	<0.001	0.40 - 0.61[3]	<0.001
COPD	0.35/0.38	<0.001	0.45	<0.001
AAA	0.44/0.44	<0.001	0.66	<0.001
RA	0.48/0.54	<0.001	0.48	<0.001
Bronchiectasis	0.24/0.42	<0.001	0.92	<0.001

[1] Two-tailed.

[2] The predicted rho values were calculated using a linear mixed-effects model incorporating subject-specific random effects and taking into account the effects of multiple visits from some individuals.

[3] The rho values are dependent on the comparator group.

Supplementary Table 3 – Ethics approvals for the cohorts included in this current study

	**Ethics committee**	**Reference number**
The ECLIPSE cohort	The study protocol was approved by the local ethics committees for all 46 participating sites in 12 countries. See Supplementary Table 4 for the full list.	SCO104960 (ECLIPSE) RM 2005/00273/05
The Nottingham COPD cohort	Nottingham Research Ethics Service	10/H0406/65
The Scotland COPD cohort	The West Glasgow Research Ethics Committee	07/SO709/46
The TAYBRIDGE cohort	East of Scotland Ethics Research Service	12/ES/0059
The Rheumatoid Arthritis cohort	The Tayside Committee on Medical Ethics	05/S1401/112
The MA3RS cohort	East of Scotland Ethics Research Service	12/ES/0068
The UKAGS cohort	East Midlands – Leicester Central Research Ethics Committee	09/H0406/119

Supplementary Table 4. Participating centers in the ECLIPSE study

Inv/Site No.	Institution & Address	IEC/IRB Committee
082272/023579	Haukeland Universitets sykehus<br>Chest department<br>Jonas Liesvei 65<br>Bergen N 5021 Norway	Regional Ethic Committee West<br>Haukeland University Hospital, N-5021 Bergen, Norway
014566/024144	P3 Research Bown Hospital<br>Churchill Drive Crofton Downs<br>Wellington 6035 New Zealand	c/- Ministry of Health<br>1-3 The Terrace<br>Level 1<br>Wellington<br>6011
136098/024146	KOPA Golnik<br>Golnik 36<br>4204 Golnik Slovenia	The National Medical Ethics Committee of the Republic of Slovenia<br>University Institute of Clinical Neurophysiology,<br>Medical Center Ljubljana,<br>Zaloška c. 7,<br>SI-1525 Ljubljana
108244/026658	Hospital Son Dureta<br>C/ Andrea Doria 55<br>Palma de Mallorca 07014 Spain	Comité ètic d´investigació clínica Illes Balears<br>Conselleria de Salut i Consum<br>Direcció General d'Avaluació i Acreditació<br>Comitè Ètic d'Investigació Clínica de les Illes Balears (CEIC-IB)<br>Camí de Jesús, 38 A<br>07011 Palma - Illes Balears
000473/023973	Aintree University Hospitals NHS Foundation Trust<br>Respiratory Research Department<br>Longmoor Lane, Ward 14a<br>Liverpool L9 7AL United Kingdom	Oxfordshire REC C<br>2nd Floor, Astral House<br>Chaucer Business Park<br>Granville Way<br>Bicester OX26 4JT
082424/023706	Cambridge Institute for Medical Research<br>Department of Medicine<br>Hills Road, Wellcome Trust / MRC Building<br>Cambridge CB2 2XY United Kingdom	Oxfordshire REC C<br>2nd Floor, Astral House<br>Chaucer Business Park<br>Granville Way<br>Bicester OX26 4JT
029855/023707	New Royal Infirmary of Edinburgh<br>Little France Crescent, Old Dalkeith Road<br>51 Little France Crescent<br>Edinburgh Midlothian EH16 4SA<br>United Kingdom	Oxfordshire REC C<br>2nd Floor, Astral House<br>Chaucer Business Park<br>Granville Way<br>Bicester OX26 4JT

023731/023974	Wythenshawe Hospital<br>Medicine Evaluation Unit<br>Southmoor Road<br>The Langley Building, North West Lung Research Centre<br>Manchester M23 9LT United Kingdom	Oxfordshire REC C<br>2nd Floor, Astral House<br>Chaucer Business Park<br>Granville Way<br>Bicester OX26 4JT
029742/024037	The Royal Free Hospital<br>Academic Unit of Respiratory Medicine<br>Pond Street<br>London NW3 2QG United Kingdom	Oxfordshire REC C<br>2nd Floor, Astral House<br>Chaucer Business Park<br>Granville Way<br>Bicester OX26 4JT
001069/024393	Institute of Phthisiatry and Pulmonology<br>Department of Pulmonology<br>10, Amosova Str<br>Kiev 03680 Ukraine	
001047/024392	Institute of Phthisiatry and Pulmonology<br>Department of Pulmonology<br>10, Amosova Str<br>Kiev 03680 Ukraine	
001071/024362	Institute of Phthisiatry and Pulmonology<br>Department of Pulmonology<br>10, Amosova Str<br>Kiev 03680 Ukraine	
001063/024364	Donetsk State Medical University<br>Department of Therapy<br>16 Illicha prospect<br>Donetsk 83003 Ukraine	
044783/023140	University of Texas Health Science Center<br>Pulmonary Diseases<br>7400 Merton Minter Blvd., (111E)<br>San Antonio, TX 78229 United States	University of Texas Health Science Center<br>7703 Floyd Curl Drive, Mail Code 7830<br>San Antonio, TX 78229-3900
077534/023146	Rhode Island Hospital<br>Division of Pulmonary, Sleep & Critical Care Medicine<br>593 Eddy Street, APC 7th Floor<br>Providence, RI 02903 United States	Lifespan Office of Research Administration<br>167 Point Street<br>Providence, RI 02903
013075/023147	Los Angeles Biomedical Research Institute at Harbor-UCLA Medical Center<br>Rehab Clinical Trials Center	John F. Wolf, MD Human Subjects Committee<br>Los Angeles Biomedical Research Institute at Harbor-UCLA Medical Center<br>1124 West Carson Street

	1124 W. Carson St., Bldg. J4<br>Torrance, CA 90502 United States	Torrance, CA 90502
008578/023354.	St. Elizabeth's Medical Center<br>Pulmonary STN-3<br>736 Cambridge Street<br>Boston, MA 02135 Unites States	Research/Human Subjects Committee<br>Caritas St. Elizabeth's Medical Center<br>Cambridge St., HOQ3<br>Boston, MA 02135
021992/023148	Pulmonary Associates of Richmond, Inc.<br>1000 Boulders Parkway, Suite 201<br>Richmond, VA 23225 United States	Goodwyn Institution Review Board<br>9380 Main Street<br>Cincinnati, OH 45242
010875/023149	Pulmonary Associates, PA<br>1112 East McDowell Road<br>Phoenix, AZ 85006 United States	Goodwyn Institution Review Board<br>9380 Main Street<br>Cincinnati, OH 45242
011553/023150	Advances in Medicine<br>42362 Bob Hope Drive<br>Rancho Mirage, CA 92270 Unites States	Western International Review Board<br>3535 Seventh Ave SW<br>Olympia, WA 98508
010094/023355	Baylor Clinic-Baylor College of Medicine<br>6620 Main Street<br>Suite 11B, 16<br>Houston, TX 77030 United States	Baylor College of Medicine IRB<br>Clinical Research Studies<br>One Baylor Plaza, Mail stop 600D<br>Houston, TX 77030
015497/023356	Dartmouth-Hitchcock Medical Center<br>Pulmonary & Critical Care Center<br>One Medical Center Drive<br>Lebanon, NH 03756 Unites States	Dartmouth-Hitchcock Medical Center<br>Committee for the Protection of Human Subjects<br>11 Rope Ferry Road #6210<br>Hanover, NH 03755
008005/023357	National Jewish Medical & Research Center<br>Weinberg Clinical Research Unit<br>1400 Jackson Street<br>Denver, CO 80206 United States	National Jewish Medical & Research Center IRB<br>1400 Jackson Street<br>Denver, CO 80206
009021/023358	University of Nebraska Medical Center<br>Pulmonary Clinical Studies Unit<br>982465 Nebraska Medical Center<br>DRC II1022<br>Omaha, NE 68198 United States	University of Nebraska Medical Center IRB<br>Academic & Research Services Bldg. 3000<br>987830 Nebraska Medical Center<br>Omaha, NE 68198

083482/023571	Yale University School of Medicine<br>Internal Medicine/Pulmonary<br>1 Gilbert Street, TAC S 441<br>New Haven, CT 06520 United States	Yale University School of Medicine<br>Human Investigation Committee<br>47 College Street, Suite 204<br>New Haven, CT 06520
015449/023359	Mayo Clinic<br>Pulmonary Clinical Research Center<br>Lanmark 2-46<br>14 – 2nd Street SW<br>Rochester, MN 55905 United States	Mayo Foundation IRB<br>201 Building, Room 4-60<br>200 First Street SW<br>Rochester, MN 55905
080801/023389	Creighton University Medical Center<br>Pulmonary & Critical Care Division<br>601 N. 30th Street, Suite 3820<br>Omaha, NE 68131 United States	Creighton University Medical Center IRB<br>2500 California Plaza<br>Omaha, NE 68178
010532/023489	University of Pittsburgh Medical Center<br>Emphysema Research Center<br>3471 5th Ave., Suite 1211<br>Pittsburgh, PA 15213 United States	University of Pittsburgh IRB<br>3500 Fifth Ave, Ground Level<br>Pittsburgh, PA 15213
021093/023390	Houston VA Medical Center<br>2002 Holcombe Blvd.<br>Pulmonary 111-1, Room 3C-220<br>Houston, TX 77030 United States	Baylor College of Medicine IRB<br>Clinical Research Studies<br>One Baylor Plaza, Mail stop 600D<br>Houston, TX 77030
008864/023392	Midwest Chest Consultants, PC<br>330 First Capital Drive, Suite 470<br>St. Charles, MO 63301 United States	Goodwyn Institution Review Board<br>9380 Main Street<br>Cincinnati, OH 45242
077533/023391	Harvard University–Brigham & Women's Hospital<br>Channing Laboratory<br>181 Longwood Ave.<br>Boston, MA 02115 United States	Brigham & Women's Hospital IRB<br>Partners Human Research Office<br>116 Huntington Ave, Suite 1002<br>Boston, MA 02116
016586/023393	University of Miami School of Medicine<br>1600 NW 10th Ave, #7064-A (R-47)<br>Miami, FL 33136	Western International Review Board<br>3535 Seventh Ave SW<br>Olympia, WA 98508
057745/023394	Johns Hopkins Asthma & Allergy Center<br>5501 Hopkins Bayview Circle, Room 3B-58	John Hopkins School of Medicine<br>Office of Human Subject Research<br>1620 McElderry St., Reed Hall<br>Suite B 130<br>Baltimore, MD 21205

	Baltimore, MD 21224 United States	
012252/023395	St. Francis Hospital & Medical Center<br>Pulmonary Medicine<br>114 Woodland Street<br>Hartford, CT 06105 United States	St. Francis Hospital & Medical Center IRB<br>Department of Research<br>114 Woodland St.<br>Hartford, CT 06105
